# Low alanine aminotransferase as a risk factor for chronic obstructive pulmonary disease in males

**DOI:** 10.1038/s41598-021-94385-0

**Published:** 2021-07-21

**Authors:** Yong Jun Choi, Do Sun Kwon, Taehee Kim, Jae Hwa Cho, Hyung Jung Kim, Min Kwang Byun, Hye Jung Park

**Affiliations:** Department of Internal Medicine, Gangnam Severance Hospital, Yonsei University College of Medicine, 211, Eonju-ro, Gangnam-gu, Seoul, 06273 Korea

**Keywords:** Risk factors, Epidemiology, Chronic obstructive pulmonary disease

## Abstract

Alanine aminotransferase (ALT) levels reflect skeletal muscle volume and general performance, which are associated with chronic obstructive pulmonary disease (COPD) development and prognosis. This study aimed to investigate ALT levels as a risk factor for COPD development. This 13-year population-based retrospective observational cohort study included 422,452 participants for analysis. We classified groups according to the baseline ALT levels (groups 1–5: ALT (IU/L) < 10; 10–19; 20–29; 30–39; and ≥ 40, respectively). The incidence of COPD was the highest in group 1, decreasing as the group number increased in males, but not in females. The Cox regression analysis in males revealed that a lower ALT level, as a continuous variable, was a significant risk factor for COPD development [univariable, hazard ratio (HR): 0.992, 95% confidence interval (CI): 0.991–0.994; multivariable, HR: 0.998, 95% CI: 0.996–0.999]. In addition, COPD was more likely to develop in the lower ALT level groups (groups 1–4; < 40 IU/L), than in the highest ALT level group (group 5; ≥ 40 IU/L) (univariable, HR: 1.341, 95% CI: 1.263–1.424; multivariable, HR: 1.097, 95% CI: 1.030–1.168). Our findings suggest that males with low ALT levels should be carefully monitored for COPD development.

## Introduction

Chronic obstructive pulmonary disease (COPD) is a chronic airway disease requiring life-long management. COPD has contributed to an enormous socioeconomic burden worldwide^1^. Early detection of COPD can lead to appropriately timed treatment, prevention of airway remodeling in early stages, and improvements in the symptoms and prognosis of COPD^2,3^. Determining risk factors predicting COPD development aids in the early detection of COPD. To date, we know that heavy smoking history, old age, male sex, and other epidemiologic factors contribute to COPD development^4,5^.

Alanine aminotransferase (ALT) levels can be easily measured from the serum and are commonly used to evaluate liver disease^6^. However, recent studies have revealed that low ALT levels predict poor health outcomes, including death, and reflect skeletal muscle volume; these are important factors representing the general condition and performance in old age^7–13^. In particular, decreased skeletal muscle volume is an important risk factor for COPD development^14,15^. Therefore, low ALT levels might adversely affect COPD development. Moreover, recent studies have revealed that liver diseases, such as non-alcoholic fatty liver disease, affect COPD development and prognosis. Therefore, high ALT levels might also adversely affect COPD^16–18^.

This study aimed to investigate the role of serum ALT levels in predicting COPD development, using a large, nationwide, population-based cohort database.

## Results

### Baseline clinical characteristics

The 422,452 participants were divided into five groups based on the serum ALT level at the time of the health check-up. The baseline characteristics of each group are described in Table 1. Most variables were associated with the ALT group. The proportion of females was higher in groups 1 and 2 (76.7% and 62.2%, respectively) and lower in groups 3, 4, and 5 (39.7%, 27.6%, and 21.7%, respectively; *P* < 0.0001). Smoking status was also significantly different according to the ALT group. The proportion of current smokers was higher in group 5 (34.2%) than in groups 1 (11.9%) and 2 (17.4%) (*P* < 0.0001). The percentage of participants who did not exercise was higher in group 1 (59.4%) than in the other groups, especially group 5 (51.7%) (*P* < 0.001). Participants in group 1 were younger and had a lower body mass index (BMI), blood pressure, hemoglobin level, fasting glucose level, and total cholesterol level than those in other groups (*P* < 0.0001).

**Table 1:** Baseline characteristics of participants according to the serum ALT levels.

		Overall	Group 1	Group 2	Group 3	Group 4	Group 5	*P*-value
Number of participants, n (%)		422,452	12,169 (2.9)	168,765 (39.9)	128,723 (30.5)	57,700 (13.7)	55,095 (13.0)	
**Categorical variables, n (%)**								
Sex	Male	229,095 (54.2)	2,834 (23.3)	63,741 (37.8)	77,648 (60.3)	41,751 (72.4)	43,121 (78.3)	< 0.0001
	Female	193,357 (45.8)	9,335 (76.7)	105,024 (62.2)	51,075 (39.7)	15,949 (27.6)	11,974 (21.7)	
Smoking status	Never smoker	268,746 (63.6)	9,725 (79.9)	122,729 (72.7)	78,430 (60.9)	30,920 (53.6)	26,942 (48.9)	< 0.0001
	Ex-smoker	35,876 (8.5)	464 (3.8)	9,821 (5.8)	12,138 (9.5)	6,582 (11.4)	6,871 (12.5)	
	Current smoker	99,825 (23.6)	1,449 (11.9)	29,285 (17.4)	32,726 (25.4)	17,533 (30.4)	18,832 (34.2)	
	Unknown	18,005 (4.3)	531 (4.4)	6,930 (4.1)	5,429 (4.2)	2,665 (4.6)	2,450 (4.4)	
Physical activity (number of times/week)	0/week	227,939 (54.0)	7,236 (59.4)	95,223 (56.4)	67,694 (52.6)	29,321 (50.8)	28,465 (51.7)	< 0.0001
	1–2/week	104,312 (24.7)	2,542 (20.9)	38,011 (22.5)	32,153 (25.0)	15,847 (27.5)	15,759 (28.6)	
	3–4/week	40,511 (9.6)	1,082 (8.9)	15,700 (9.3)	12,916 (10.0)	5,780 (10.0)	5,033 (9.1)	
	5–6/week	11,144 (2.6)	276 (2.3)	4,572 (2.7)	3,606 (2.8)	1,448 (2.5)	1,242 (2.3)	
	Almost every day	25,817 (6.1)	639 (5.3)	10,303 (6.1)	8,551 (6.6)	3,450 (6.0)	2,874(5.2)	
	Unknown	12,729 (3.0)	394 (3.2)	4,956 (3.0)	3,803 (3.0)	1,854 (3.2)	1,722 (3.1)	
**Continuous variables, mean ± standard deviation**								
Age		49.7 ± 7.1	47.7 ± 6.7	49.6 ± 7.1	50.4 ± 7.1	49.9 ± 7.0	49.0 ± 6.8	< 0.0001
Comorbidities	CCI score	0.4 ± 0.7	0.3 ± 0.7	0.4 ± 0.7	0.4 ± 0.7	0.4 ± 0.8	0.4 ± 0.8	< 0.0001
BMI (kg/m^2^)		24.1 ± 2.9	22.7 ± 2.7	23.3 ± 2.8	24.2 ± 2.8	24.8 ± 2.8	25.4 ± 3.0	< 0.0001
Systolic blood pressure (mmHg)		125.8 ± 17.7	120.2 ± 17.1	123.1 ± 17.3	126.8 ± 17.6	128.7 ± 17.5	130.1 ± 17.4	< 0.0001
Diastolic blood pressure (mmHg)		79.4 ± 11.7	75.6 ± 11.4	77.4 ± 11.5	80.0 ± 11.6	81.5 ± 11.6	82.5 ± 11.6	< 0.0001
Hemoglobin (g/dL)		14.0 ± 1.5	12.9 ± 1.6	13.5 ± 1.5	14.1 ± 1.4	14.5 ± 1.4	14.7 ± 1.4	< 0.0001
Fasting blood glucose (mg/dL)		97.4 ± 33.7	91.7 ± 28.5	94.1 ± 30.7	97.9 ± 34.1	100.6 ± 36.2	104.4 ± 38.2	< 0.0001
Total cholesterol (mg/dL)		200.3 ± 38.3	187.5 ± 37.0	195.2 ± 36.3	202.1 ± 37.6	205.9 ± 39.2	208.6 ± 41.9	< 0.0001
Liver function test	AST (IU/L)	26.6 ± 17.1	17.9 ± 19.8	20.6 ± 6.0	25.2 ± 7.5	30.0 ± 10.5	46.2 ± 35.5	< 0.0001
	ALT (IU/L)	26.2 ± 21.3	7.8 ± 1.4	15.0 ± 2.7	23.9 ± 2.8	33.7 ± 2.8	61.8 ± 40.3	< 0.0001
	ɤGT (IU/L)	36.9 ± 49.9	16.0 ± 15.1	21.1 ± 17.1	33.5 ± 30.9	48.8 ± 46.5	85.1 ± 101.5	< 0.0001

*ALT* alanine aminotransferase, *AST* aspartate transaminase, *BMI* body mass index, *CCI* Charlson’s comorbidity index, *ɤGT* gamma-glutamyl transferase.

### ALT level and incidence of COPD development

**Figure 1:**
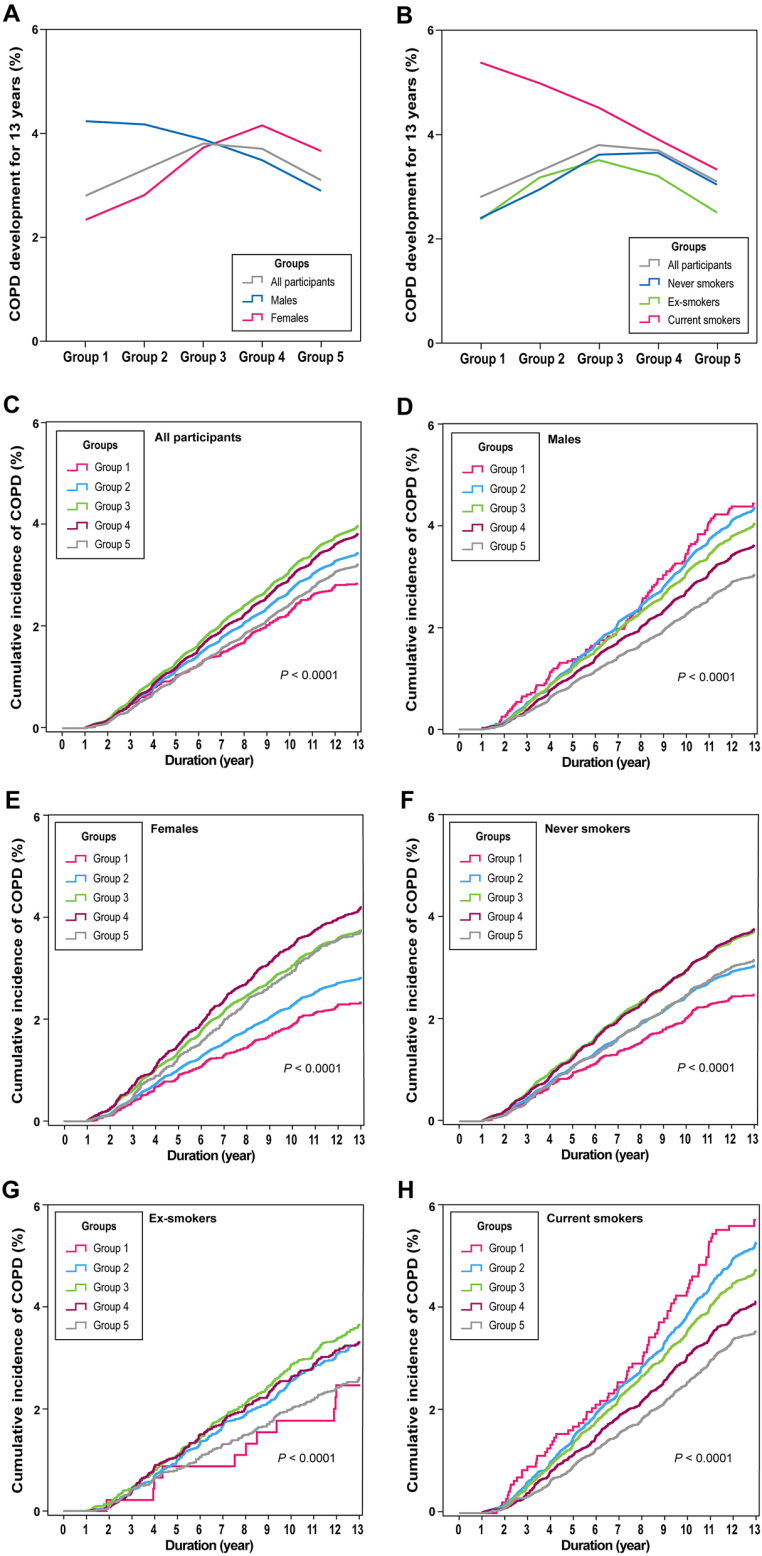
Prevalence and incidence of COPD development over 13 years. Prevalence of COPD development over 13 years according to sex **(A)** and smoking status **(B)**. Kaplan–Meier curves for the cumulative incidence of COPD development over 13 years according to the ALT group in all participants **(C)**, males **(D)**, females **(E)**, never smokers **(F)**, ex-smokers **(G)**, and current smokers **(H)**. *ALT* alanine aminotransferase, *COPD* chronic obstructive pulmonary disease. Adobe Illustrator version Creative Cloud was used to arrange the graphs and to add legends. (http://www.adobe.com/au/products/illustrator.html).

The prevalence of newly developed COPD in the study population over the 13-year period was 3.5% (n = 14,656). Comparing the five groups according to the serum ALT level, the prevalence of newly developed COPD was 2.8%, 3.3%, 3.8%, 3.7%, and 3.1%, respectively (*P* < 0.0001; Fig. 1A). There was also no consistent correlation between the ALT group and COPD development in the cumulative incidence (Fig. 1C).

We conducted a subgroup analysis according to sex and smoking status. In male participants, COPD development was the most prevalent in group 1, decreasing as the group number increased (4.2%, 4.2%, 3.9%, 3.5%, and 2.9%, respectively; *P* < 0.0001). Conversely, in female participants, COPD development was the most prevalent in group 4, decreasing as the group number decreased, except for group 5 (2.3% in group 1, 2.8% in group 2, 3.7% in group 3, 4.1% in group 4, and 3.7% in group 5; *P* < 0.0001). On comparing the cumulative incidence of COPD, the steepness of the slopes of the Kaplan–Meier curves in male participants increased as the group number decreased; however, these values were not sequential in female participants (Fig. 1D,E).

Among the subgroups divided according to smoking status, a negative correlation with COPD prevalence was only observed in the current smoker subgroup (5.4% in group 1, 5.0% in group 2, 4.5% in group 3, 3.9% in group 4, and 3.3% in group 5; *P* < 0.0001). In the other subgroups (never smokers and ex-smokers), the results were similar to the patterns observed in female participants (Fig. 1B). The steepness of the slopes of the Kaplan–Meier curves for cumulative COPD development in current smokers increased as the group number decreased; however, these values were not sequential in the other subgroups (Fig. 1F–H).

### ALT level as a risk factor for COPD development

The univariable and multivariable Cox regression analyses revealed that old age, male sex, smoking, comorbidities, lack of exercise, low systolic blood pressure, and lower ALT levels were significant risk factors for COPD development [hazard ratio (HR): 0.998, 95% confidence interval (CI): 0.997–0.999, *P* < 0.0001; Supplementary Table 1]. To assess whether the ALT level was related to other variables that are already well-established risk factors for COPD development, correlation coefficients between each variable were analyzed and drawn as a graph (Supplementary Fig. 1A). ALT showed a strong association with other liver function tests (aspartate aminotransferase [AST], gamma-glutamyl transferase [ɤGT]); however, it did not show an apparent association with other risk factors for COPD development at baseline. Male sex was highly associated with current smoking; therefore, subgroup analysis was conducted according to sex and smoking status to assess the specific factors that more strongly explained the association between low ALT and COPD.

In males, lower ALT level was a significant risk factor for COPD development according to the multivariable Cox regression analysis (HR: 0.998, 95% CI: 0.996–0.999, *P* = 0.0001; Table 2). However, in females, the ALT level was not a significant risk factor for COPD development (HR: 1.000, 95% CI: 0.999–1.002, *P* = 0.8068; Supplementary Table 2). In both males and females, the correlation coefficients between each variable were also analyzed to identify the link between the ALT level and other risk factors for COPD. The ALT level did not show an apparent association with other risk factors for COPD development (Supplementary Fig. 1B,C).

**Table 2:** Cox regression analyses for COPD development in males.

Variable	Univariable analysis			Multivariable analysis		
	HR	95% CI	*P*-value	HR	95% CI	*P*-value
**Smoking (never smoker vs.)**						
Ex-smoker	0.934	0.872–1.001	0.0549	1.117	1.041–1.199	0.0022
Current smoker	1.275	1.216–1.336	< 0.0001	1.539	1.465–1.617	< 0.0001
**Physical activity (0/week vs.)**						
1–2/week	0.652	0.619–0.686	< 0.0001	0.772	0.732–0.814	< 0.0001
3–4/week	0.590	0.544–0.640	< 0.0001	0.675	0.621–0.734	< 0.0001
5–6/week	0.708	0.615–0.814	< 0.0001	0.763	0.662–0.880	0.0002
Almost every day	1.021	0.939–1.112	0.6234	0.852	0.781–0.929	0.0003
Age	1.101	1.097–1.104	< 0.0001	1.099	1.095–1.102	< 0.0001
Comorbidities (CCI score)	1.274	1.248–1.300	< 0.0001	1.203	1.177–1.231	< 0.0001
BMI (kg/m^2^)	0.934	0.926–0.941	< 0.0001	0.964	0.956–0.972	< 0.0001
Systolic blood pressure (mmHg)	1.001	1.000–1.002	0.1457			
Diastolic blood pressure (mmHg)	0.995	0.993–0.997	< 0.0001	0.994	0.992–0.996	< 0.0001
Hemoglobin (g/dL)	0.907	0.890–0.924	< 0.0001	1.013	0.993–1.033	0.2164
Fasting blood glucose (mg/dL)	0.999	0.998–1.000	0.0111	0.998	0.997–0.999	< 0.0001
Total cholesterol (mg/dL)	0.998	0.997–0.999	< 0.0001	0.999	0.999–1.000	0.0093
AST (IU/L)	0.999	0.998–1.001	0.3200			
ALT (IU/L)	0.992	0.991–0.994	< 0.0001	0.998	0.996–0.999	0.0001
ɤGT (IU/L)	1.000	0.999–1.000	0.1404			

*ALT* alanine aminotransferase, *AST* aspartate aminotransferase, *BMI* body mass index, *CCI* Charlson’s comorbidity index, *CI* confidence interval, *COPD* chronic obstructive pulmonary disease, *HR* hazard ratio, *ɤGT* gamma-glutamyl transferase.

A subgroup analysis according to smoking status revealed a similar pattern. In the current smoker subgroup, lower ALT level was a significant risk factor for COPD development (HR: 0.996, 95% CI: 0.994–0.998, *P* = 0.0002; Supplementary Table 3). However, the ALT level was not a significant risk factor in never smokers (HR: 1.000, 95% CI: 0.998–1.001, *P* = 0.4678; Supplementary Table 4).

**Figure 2:**
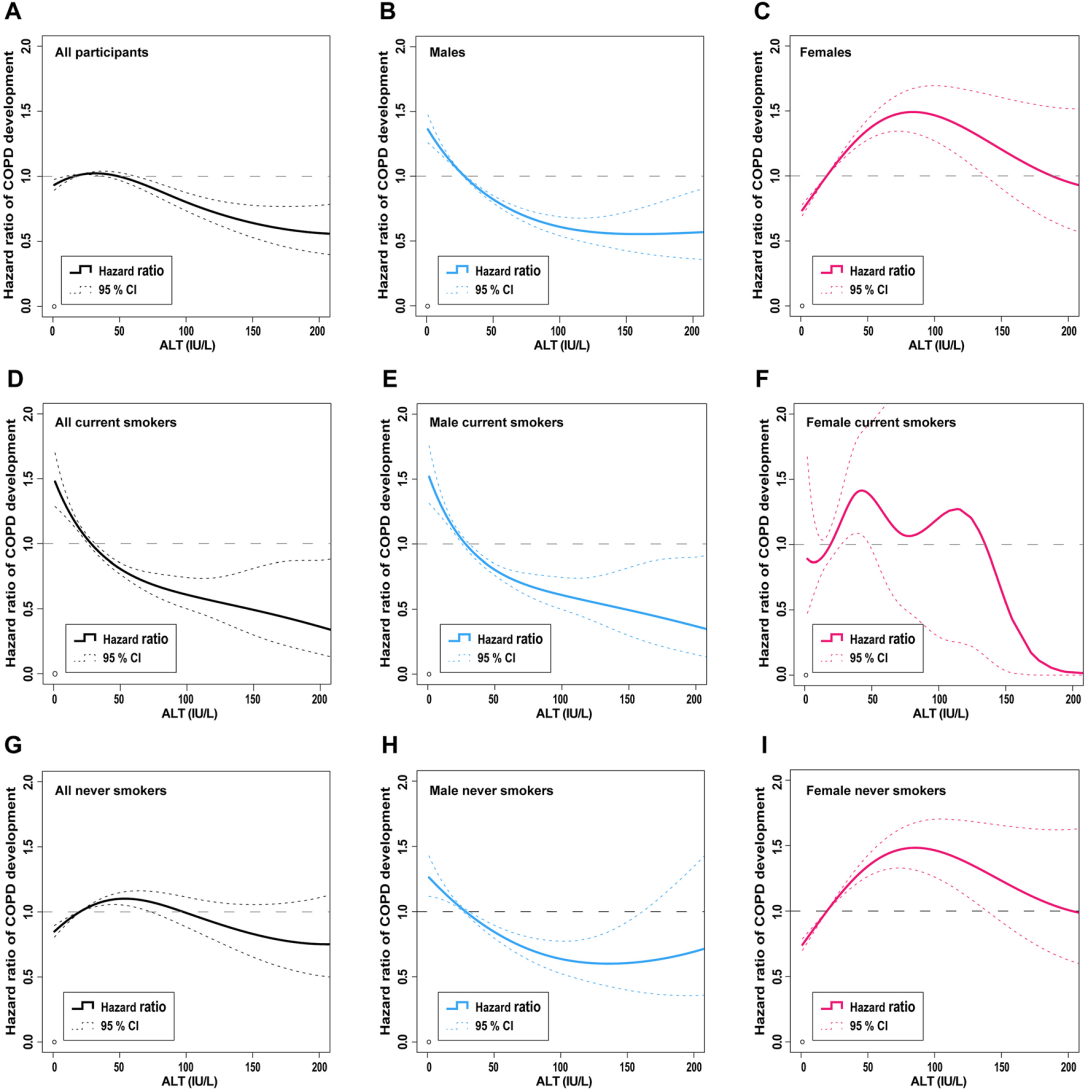
Spline curves of the hazard ratio for COPD development. Spline curves are shown for all participants **(A)**, males **(B)**, females **(C)**, all current smokers **(D)**, male current smokers **(E)**, female current smokers **(F)**, all never smokers **(G)**, male never smokers **(H)**, and female never smokers **(I)**. *ALT* alanine aminotransferase, *CI* confidence interval, *COPD* chronic obstructive pulmonary disease. Adobe Illustrator version Creative Cloud was used to arrange the graphs and to add legends (http://www.adobe.com/au/products/illustrator.html).

Spline curves between ALT and the HR for COPD development were compared according to smoking status in each sex group (Fig. 2). As with the previous results, in all participants and females, the curves did not show a consistent correlation between the ALT levels and the HR for COPD development (Fig. 2A,C, respectively). By contrast, the curves in males showed a negative correlation between ALT and the HR of COPD development (Fig. 2B).

In the current smoker subgroup, the curves of all and male current smokers showed a negative correlation between ALT and the HR of COPD development (Fig. 2D,E, respectively); by contrast, the curves of female current smokers did not show a consistent correlation (Fig. 2F). In never smokers, only the curve of male never smokers showed a negative correlation; the other subgroups did not show a consistent correlation (Fig. 2G–I, respectively). The results suggest that ALT is a significant risk factor in males rather than in current smokers.

In males, we redefined groups 1–4 and group 5 as low and high ALT groups, respectively. Comparisons between these two groups are described in Supplementary Table 5. As with the baseline characteristics of all participants, most variables were associated with the ALT group.

**Figure 3:**
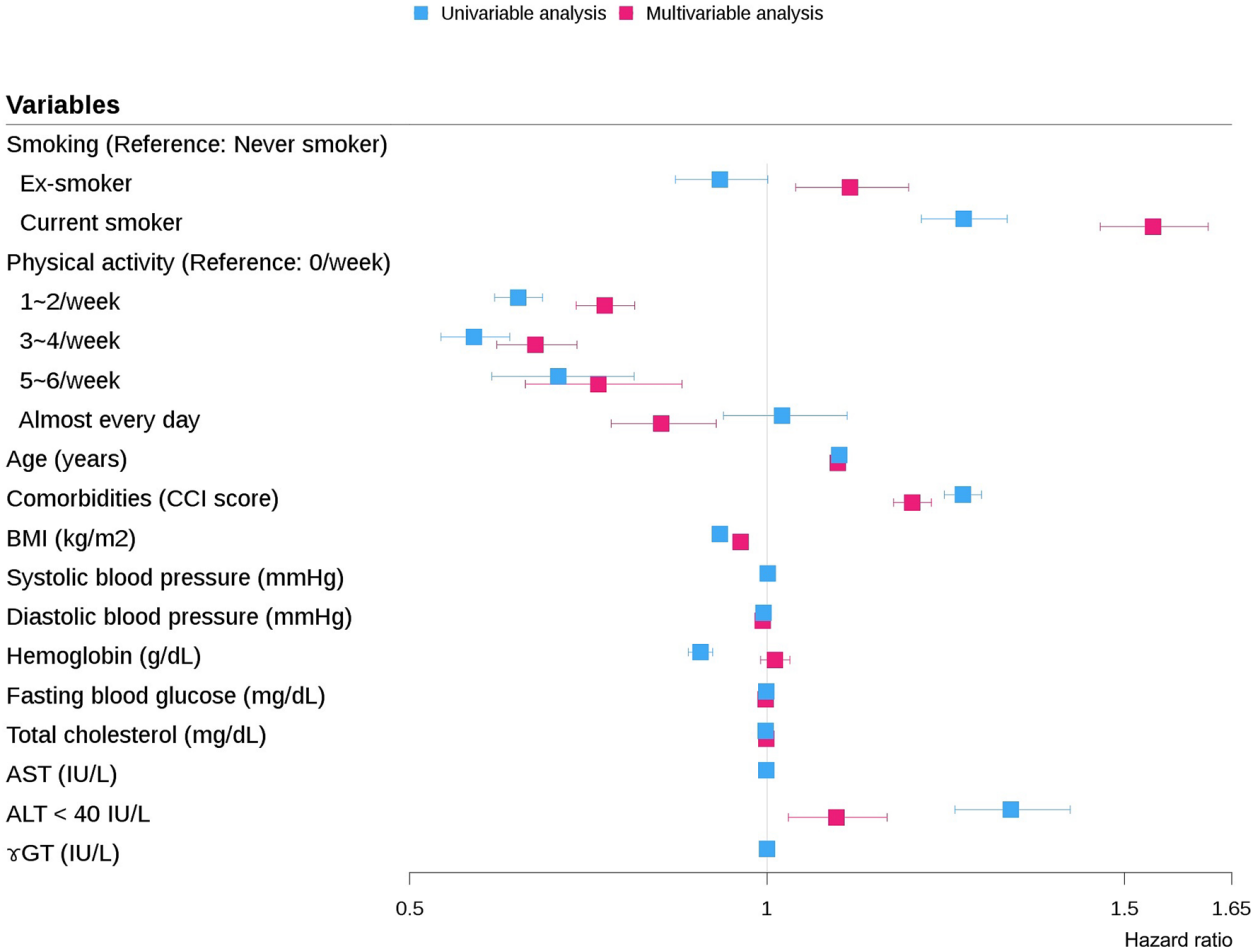
Hazard ratios for COPD development in males. *AST* aspartate aminotransferase, *ALT* alanine aminotransferase, *BMI* body mass index, *CCI* Charlson’s comorbidity index, *COPD* chronic obstructive pulmonary disease, *ɤGT* gamma-glutamyl transferase.

**Figure 4:**
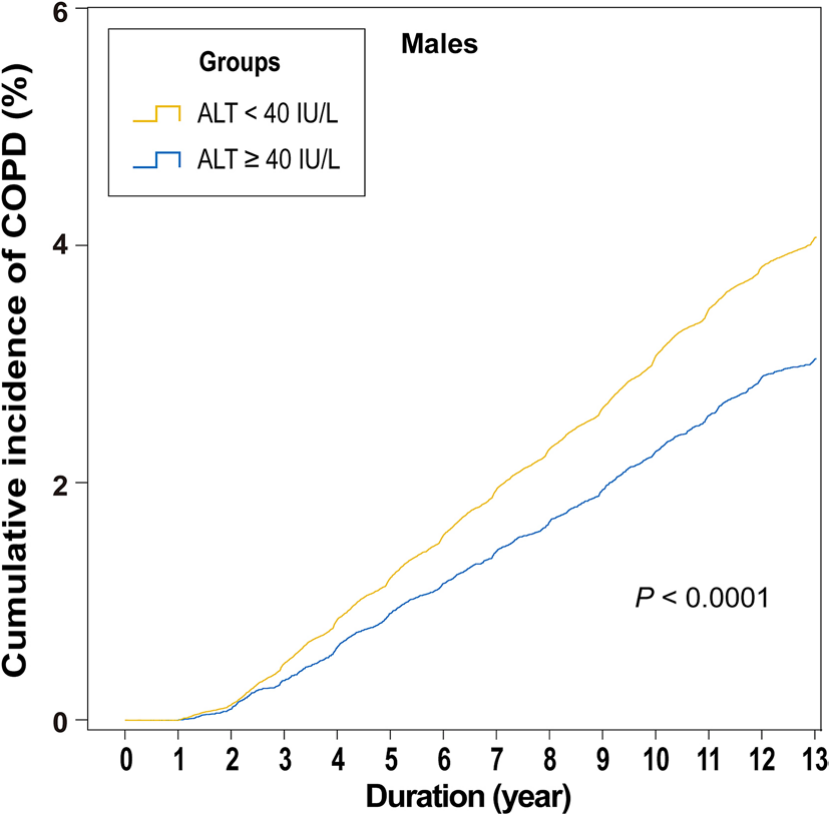
Kaplan–Meier curves of the cumulative COPD development in males. *ALT* alanine aminotransferase, *COPD* chronic obstructive pulmonary disease.

Additional analyses showed that in males, low ALT level was a significant risk factor for COPD development on both univariable (HR: 1.341, 95% CI: 1.263–1.424, *P* < 0.0001) and multivariable Cox regression (HR: 1.097, 95% CI: 1.030–1.168, *P* = 0.0042) analyses (Fig. 3). The Kaplan–Meier curves for the cumulative COPD development in the two groups also showed a significant difference. In the low ALT group, the cumulative incidence of COPD was higher than that in the high ALT group (3.9% vs. 2.9%, *P* < 0.0001, Fig. 4).

## Discussion

To our knowledge, this is the first study to find that a lower serum ALT level is a risk factor for COPD development in males. We believe that ALT levels reflect the overall health status, as described previously^10^. Therefore, the HR of the ALT level became weaker after adjusting for other risk factors. However, low ALT level remained an independent risk factor for COPD development after adjusting for other factors.

We also observed that the effects of ALT level on COPD development differed according to sex. Previous studies have described the association between ALT levels and obesity. Fat accumulation accelerates the chronological aging of liver cells, increasing the vulnerability of the liver cells to oxidative stress and energy metabolic pressure, thus increasing liver enzyme levels^19–21^. However, this process can differ in males and females^22,23^. Since sex hormones affect fat and muscle distributions, the subcutaneous fat storage capacity is lower in males than in females. Therefore, in males, fat accumulates more in other tissues, including the viscera and liver, rather than in subcutaneous tissue, which may lead to greater fat accumulation in the liver in the early stages of obesity. As a result, serum ALT levels may be elevated even in the early stages of obesity^21,24–27^. Therefore, the ALT level in males may have a stronger relationship with fat accumulation associated with general nutrition, performance scales, and health status compared to that in females. This might explain why the effects of ALT level on COPD development are more substantial in males than in females. However, further studies are needed to establish the exact relationship between the ALT level and COPD.

The effects of the ALT level on COPD development were more prominent in male current smokers than in male never smokers. The relationship between smoking status and ALT level is also unclear. Breitling et al.^28^ implied a trend towards a significant negative association between smoking intensity and serum ALT levels in non-drinkers in a cross-sectional study. Most other studies have also observed an inverse association or the lack of an association between smoking and ALT levels^28–31^. However, it is known that males and current smokers are more susceptible to developing COPD. We surmise that the effect of the ALT level on COPD development in male current smokers was accentuated in these vulnerable subgroups.

According to a recent literature review, COPD is underdiagnosed in 65–80% of participants with chronic airflow limitations^32^. To control the disease effectively, the early diagnosis and treatment of COPD are vital for preventing airway remodeling in the early stages^2,3^. Additionally, the ALT level is a commonly performed test that is inexpensive, easy to obtain from serum, and included in most basic chemical tests. Therefore, this study suggests the careful monitoring of males with low ALT levels, which may be helpful in the early detection of COPD and improved COPD prognosis in these individuals. Furthermore, Lasman et al. reported that low ALT levels are associated with mortality in patients with COPD with a history of exacerbation^33^. Therefore, the careful monitoring of patients with COPD with low ALT levels may also help predict the prognosis.

Our study had several limitations. First, we divided the participants into five groups based on ALT intervals of 10 IU/L, in alignment with previous studies, because there are no reference cut-off values for low ALT levels^12^. Second, the diagnosis of COPD in our study was based on International Classification of Disease, 10th edition (ICD-10) codes and prescribed medication data from the Korean National Health Insurance Service (NHIS) database, without pulmonary function testing. Similarly, we also defined liver disease based on ICD-10 codes. Despite these limitations, our study had several strengths. Above all, it was the first study, to our knowledge, to investigate the relationship between serum ALT levels and COPD development. Moreover, it was a long-term cohort study, which allowed us to analyze the risk factors potentially affecting COPD development. Finally, the results had high statistical power owing to the large-scale analysis involving 422,452 participants.

## Conclusions

The current results suggest that low serum ALT levels may play a significant role in COPD development in males. Therefore, clinicians should carefully monitor male patients with low ALT levels for COPD development.

## Methods

### Study design and population

This retrospective cohort study was conducted in a health check-up cohort population from the Korean NHIS database. In South Korea, populations aged 40–79 years participated in the biennial national health screening program covered by the Korean National Health Insurance cooperation. This cohort group is a simple random sample representing 10% of the 5.15 million populations who participated in the national health screening program from 2002 to 2003.

**Figure 5:**
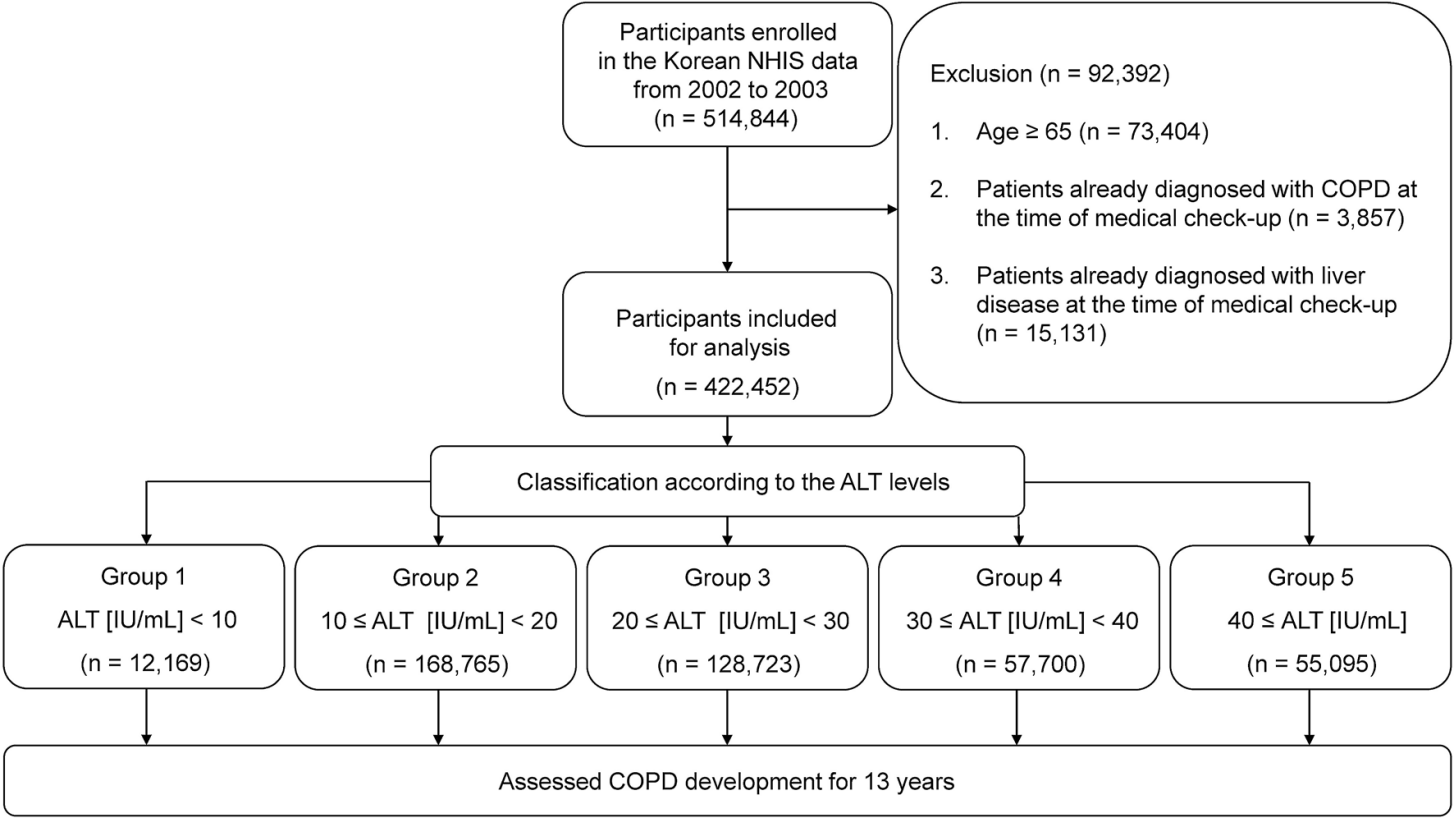
Study flowchart. *ALT* alanine aminotransferase, *COPD* chronic obstructive pulmonary disease, *NHIS* National Health Insurance Survey.

Among the 514,844 participants, 73,404 participants aged above 65 years were excluded to reduce the confounding effects of extreme old age on the development of COPD; 3857 were excluded owing to a diagnosis of COPD at the time of the health check-up; and 15,131 were excluded due to diagnoses of liver disease such as viral liver disease, non-alcoholic fatty liver disease, and hepatocellular carcinoma at the time of the health check-up, to limit the confounding effects of liver disease on ALT levels. Finally, 422,452 participants were included in the analysis (Fig. 5).

Participants were divided into five groups, based on serum ALT levels at baseline: group 1, ALT (IU/L) < 10; group 2, 10 ≤ ALT < 20; group 3, 20 ≤ ALT < 30; group 4, 30 ≤ ALT < 40; and group 5, ALT ≥ 40. COPD development was retrospectively compared among all groups for 13 years.

### Parameters included in data collection

The basic demographic data included the participant’s age and sex, and insurance claim data included the dates of hospital visits, diagnostic codes encoded by the ICD-10, and prescriptions. The Charlson’s comorbidity index was calculated based on comorbidities using ICD-10 codes^34^. Health check-up data provided the results of the health check-ups conducted in 2002 or 2003 and included the results of BMI (kg/m^2^) and systolic and diastolic blood pressure (mmHg) measurements, as well as hemoglobin (g/dL), fasting blood glucose (mg/dL), total cholesterol (mg/dL), AST (IU/L), ALT (IU/L), and ɤGT (IU/L) levels. Moreover, health check-up data included a self-report questionnaire, which the participants answered during the health check-up, providing information regarding smoking status (never smoker, ex-smoker, and current smoker) and physical activity (0, 1–2, 3–4, 5–6 times/week, and almost every day).

### Definition of COPD development

As the NHIS database does not include data from pulmonary function tests, we defined COPD development based on the diagnostic codes of the ICD-10 and prescriptions contained in the insurance claim data, as suggested by previous studies^35–37^. COPD development was determined when all the following criteria were met: (1) age above 40 years; (2) ICD-10 diagnostic code of COPD or emphysema (J43.0x–J44.x, except J43.0, as the primary or secondary diagnosis [within the fourth position]); (3) the use of COPD medications, including a muscarinic antagonist, beta-2 agonist, inhaled corticosteroid, phosphodiesterase-4 inhibitor, or methylxanthine; and (4) first diagnosis of COPD in the follow-up period.

### Definition of liver disease

Liver diseases were defined using diagnostic codes. The following ICD-10 codes were used in this study to exclude the effects of these underlying liver diseases on ALT levels: K70.x–K77.x, disease of liver; B15.x–B19.x, viral hepatitis; E83.0, Wilson disease; and C22.0, liver cell carcinoma.

### Statistical analysis

To compare baseline characteristics across groups, chi-squared tests were used for categorical variables, and paired t-test or one-way analyses of variance with Bonferroni post-hoc tests were used for continuous variables. Univariable and multivariable Cox regression analyses were performed to evaluate the HR of each variable for COPD development. A subgroup analysis was performed according to sex and smoking status. Kaplan–Meier curves were drawn to evaluate the cumulative incidence of COPD among the groups according to serum ALT level. *P-*values < 0.05 were considered to indicate statistical significance. Statistical analyses were conducted using R (version 3.3.3; R Foundation for Statistical Computing, Vienna, Austria) software. Furthermore, spline curves of the HR for COPD development were drawn using the pspline R package, and correlation coefficient plots were drawn using the corrplot R package.

### Ethics approval and consent to participate

This study used NHIS-NSC data (NHIS-2019-2-183) provided by the NHIS. The study was approved by the Institutional Review Board of Gangnam Severance Hospital (number: 3-2019-0123). As the data from NIHS contained no personal information, the need for informed consent was waived. The Institutional Review Board of Gangnam Severance Hospital approved the waiver of the informed consent. All data were collected in accordance with the amended Declaration of Helsinki. The authors declare no conflict of interest with NHIS.

## Supplementary Information

Supplementary Information.

## Data Availability

The data that support the findings of this study are available from NHIS. Restrictions apply to the availability of these data, which were used under license for this study. Data are available at URL: https://nhiss.nhis.or.kr with the permission of NHIS.

## Abbreviations

ALT: Alanine aminotransferase
AST: Aspartate aminotransferase
BMI: Body mass index
CI: Confidence interval
COPD: Chronic obstructive pulmonary disease
ɤGT: Gamma-glutamyl transferase
HR: Hazard ratio
ICD-10: International Classification of Disease, 10th edition
NHIS: National Health Insurance Service
