# Supplementary material for: Low alanine aminotransferase as a risk factor for chronic obstructive pulmonary disease in males
Source: Sci Rep. 2021 Jul 21;11:14829. doi: 10.1038/s41598-021-94385-0 (PMC8295341; doi:10.1038/s41598-021-94385-0)
Supplement: Supplementary file 1 — Supplementary Information. [file 41598_2021_94385_MOESM1_ESM.doc]

**SUPPLEMENTARY INFORMATION**

**TITLE: Low alanine aminotransferase as a risk factor for chronic obstructive pulmonary disease in males**

**Authors’ full names:** Yong Jun Choi, MD; Do Sun Kwon, MD; Taehee Kim, MD; Jae Hwa Cho, MD, PhD; Hyung Jung Kim, MD, PhD; Min Kwang Byun, MD, PhD; Hye Jung Park, MD, PhD

**Authors’ affiliation:** Department of Internal Medicine, Gangnam Severance Hospital, Yonsei University College of Medicine, Seoul, Korea

**Supplementary Figure 1***-* Correlation coefficient plot of variables associated with COPD development

*
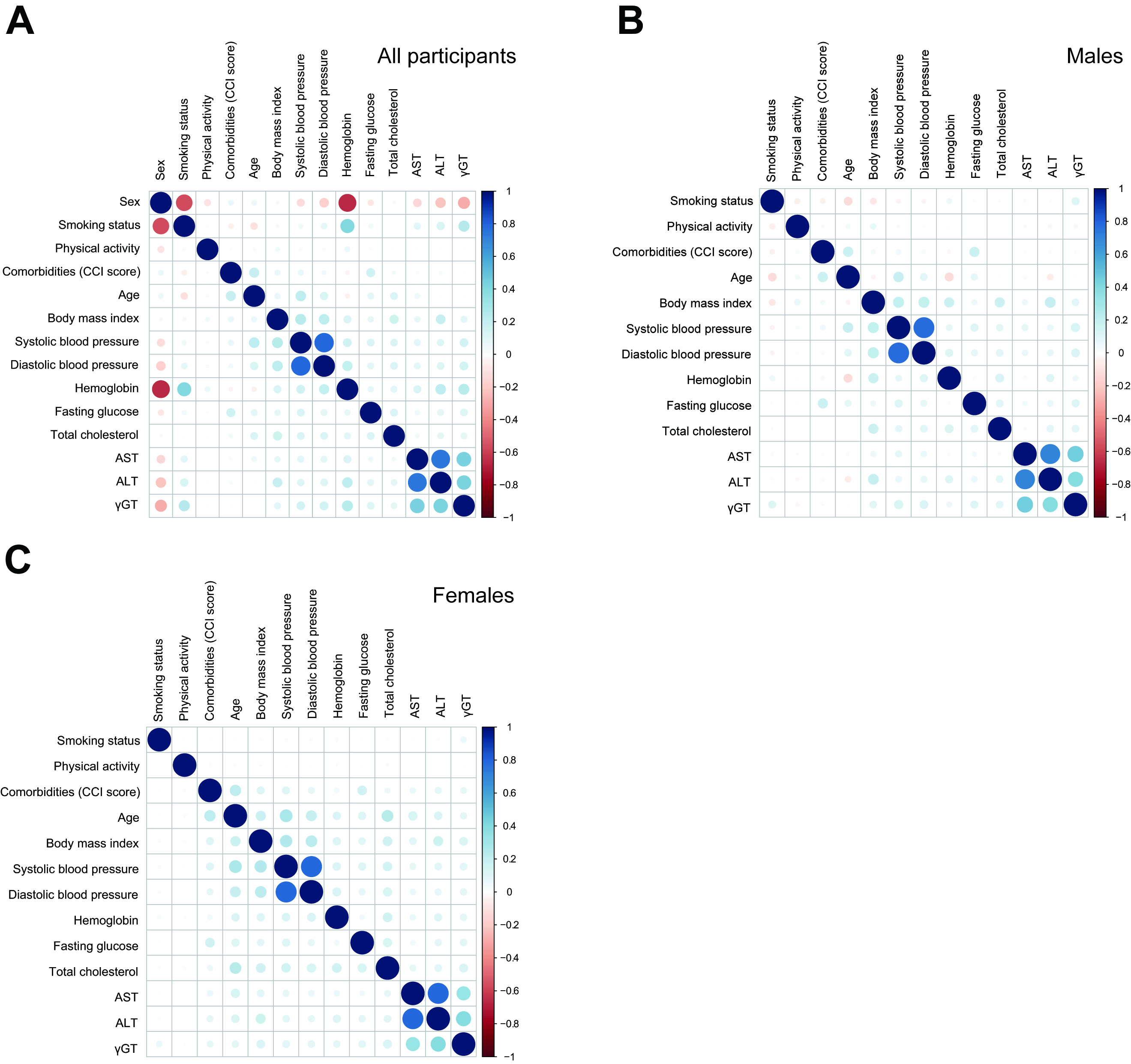
*

COPD, chronic obstructive pulmonary disease; CCI, Charlson’s comorbidity index; AST, aspartate aminotransferase; ALT, alanine aminotransferase; ɤGT, gamma-glutamyl transferase

**Supplementary Table 1-****Cox regression analyses for COPD development in all participants**

| **Variable** | **Univariable analysis** | | | **Multivariable analysis** | | |
| --- | --- | --- | --- | --- | --- | --- |
|  | **HR** | **95% CI** | ***P*-value** | **HR** | **95% CI** | ***P*-value** |
| **Sex (Male vs.)** |  | | | | | |
| **Female** | 0.853 | 0.825–0.881 | <0.0001 | 0.861 | 0.820–0.905 | <0.0001 |
| **Smoking (Never smoker vs.)** |  | | | | | |
| **Ex-smoker** | 0.981 | 0.922–1.044 | 0.5510 | 1.107 | 1.035–1.185 | 0.0033 |
| **Current smoker** | 1.374 | 1.324–1.425 | <0.0001 | 1.578 | 1.508–1.652 | <0.0001 |
| **Physical activity (0/week vs.)** |  | | | | | |
| **1**–**2/week** | 0.719 | 0.690–0.749 | <0.0001 | 0.780 | 0.747–0.814 | <0.0001 |
| **3**–**4/week** | 0.643 | 0.603–0.686 | <0.0001 | 0.693 | 0.649–0.740 | <0.0001 |
| **5**–**6/week** | 0.713 | 0.637–0.798 | <0.0001 | 0.735 | 0.656–0.824 | <0.0001 |
| **Almost every day** | 1.038 | 0.973–1.107 | 0.2600 | 0.866 | 0.811–0.925 | <0.0001 |
| **Age** | 1.092 | 1.090–1.095 | <0.0001 | 1.092 | 1.090–1.095 | <0.0001 |
| **Comorbidities (CCI score)** | 1.271 | 1.252–1.289 | <0.0001 | 1.202 | 1.183–1.222 | <0.0001 |
| **BMI (kg/m^2^)** | 0.994 | 0.989–1.000 | 0.0377 | 0.993 | 0.9872–0.999 | 0.0216 |
| **Systolic blood pressure (mmHg)** | 1.005 | 1.004–1.006 | <0.0001 | 0.997 | 0.996–0.998 | <0.0001 |
| **Diastolic blood pressure (mmHg)** | 1.002 | 1.000–1.003 | 0.0117 |  | | |
| **Hemoglobin (g/dL)** | 1.027 | 1.017–1.038 | <0.0001 | 1.011 | 0.996–1.026 | 0.1530 |
| **Fasting blood glucose (mg/dL)** | 1.000 | 1.000–1.001 | 0.6154 |  | | |
| **Total cholesterol (mg/dL)** | 1.000 | 0.999–1.000 | 0.4862 |  |  |  |
| **AST (IU/L)** | 1.002 | 1.001–1.002 | <0.0001 |  |  |  |
| **ALT (IU/L)** | 0.999 | 0.998–1.000 | 0.0077 | 0.998 | 0.997–0.999 | <0.0001 |
| **ɤGT (IU/L)** | 1.001 | 1.000–1.001 | 0.0001 | 1.000 | 1.000–1.001 | 0.1143 |
| ALT, alanine aminotransferase; AST, aspartate aminotransferase; BMI, body mass index; CCI, Charlson’s comorbidity index; CI, confidence interval; COPD, chronic obstructive pulmonary disease; HR, hazard ratio; ɤGT, gamma-glutamyl transferase | | | | | | |

**Supplementary Table 2- Cox regression analyses for COPD development in females**

| **Variable** | **Univariable analysis** | | | **Multivariable analysis** | | |
| --- | --- | --- | --- | --- | --- | --- |
|  | **HR** | **95% CI** | ***P*-value** | **HR** | **95% CI** | ***P*-value** |
| **Smoking (Never smoker vs.)** |  | | | | | |
| **Ex-smoker** | 0.919 | 0.692–1.221 | 0.5600 | 1.050 | 0.790–1.396 | 0.7366 |
| **Current smoker** | 2.080 | 1.845–2.345 | <0.0001 | 2.074 | 1.837–2.341 | <0.0001 |
| **Physical activity (0/week vs.)** |  | | | | | |
| **1**–**2/week** | 0.757 | 0.705–0.814 | <0.0001 | 0.838 | 0.779–0.902 | <0.0001 |
| **3**–**4/week** | 0.672 | 0.604–0.749 | <0.0001 | 0.753 | 0.675–0.839 | <0.0001 |
| **5**–**6/week** | 0.663 | 0.548–0.802 | <0.0001 | 0.705 | 0.582–0.855 | 0.0004 |
| **Almost every day** | 1.021 | 0.925–1.127 | 0.6790 | 0.882 | 0.798–0.975 | 0.0140 |
| **Age** | 1.085 | 1.081–1.088 | <0.0001 | 1.080 | 1.076–1.084 | <0.0001 |
| **Comorbidities (CCI score)** | 1.283 | 1.256–1.310 | <0.0001 | 1.202 | 1.175–1.230 | <0.0001 |
| **BMI (kg/m^2^)** | 1.059 | 1.051–1.066 | <0.0001 | 1.032 | 1.023–1.040 | <0.0001 |
| **Systolic blood pressure (mmHg)** | 1.008 | 1.007–1.009 | <0.0001 |  | | |
| **Diastolic blood pressure (mmHg)** | 1.008 | 1.006–1.010 | <0.0001 | 0.997 | 0.995–0.999 | 0.0059 |
| **Hemoglobin (g/dL)** | 1.095 | 1.072–1.119 | <0.0001 | 1.039 | 1.015–1.063 | 0.0012 |
| **Fasting blood glucose (mg/dL)** | 1.001 | 1.000–1.002 | 0.0022 | 0.999 | 0.998–0.999 | 0.0015 |
| **Total cholesterol (mg/dL)** | 1.002 | 1.002–1.003 | <0.0001 | 0.999 | 0.998–0.999 | 0.0001 |
| **AST (IU/L)** | 1.003 | 1.002–1.004 | <0.0001 |  | | |
| **ALT (IU/L)** | 1.003 | 1.002–1.004 | <0.0001 | 1.000 | 0.999–1.002 | 0.8068 |
| **ɤGT (IU/L)** | 1.003 | 1.002–1.003 | <0.0001 | 1.001 | 1.000–1.002 | 0.0046 |
| ALT, alanine aminotransferase; AST, aspartate aminotransferase; BMI, body mass index; CCI, Charlson’s comorbidity index; CI, confidence interval; COPD, chronic obstructive pulmonary disease; HR, hazard ratio; ɤGT, gamma-glutamyl transferase | | | | | | |

**Supplementary Table 3- Cox regression analyses for COPD development in current smokers**

| **Variable** | **Univariable analysis** | | | **Multivariable analysis** | | |
| --- | --- | --- | --- | --- | --- | --- |
|  | **HR** | **95% CI** | ***P*-value** | **HR** | **95% CI** | ***P*-value** |
| **Sex (Male vs.)** |  | | | | | |
| **Female** | 1.515 | 1.342–1.710 | <0.0001 | 1.108 | 0.970–1.266 | 0.1295 |
| **Physical activity (0/week vs.)** |  | | | | | |
| **1**–**2/week** | 0.606 | 0.564–0.652 | <0.0001 | 0.742 | 0.688–0.801 | <0.0001 |
| **3**–**4/week** | 0.582 | 0.516–0.657 | <0.0001 | 0.681 | 0.600–0.772 | <0.0001 |
| **5**–**6/week** | 0.770 | 0.622–0.952 | 0.0156 | 0.838 | 0.674–1.042 | 0.1116 |
| **Almost every day** | 1.060 | 0.936–1.200 | 0.3569 | 0.862 | 0.759–0.979 | 0.0224 |
| **Age** | 1.113 | 1.109–1.118 | <0.0001 | 1.105 | 1.101–1.110 | <0.0001 |
| **Comorbidities (CCI score)** | 1.292 | 1.255–1.331 | <0.0001 | 1.209 | 1.170–1.249 | <0.0001 |
| **BMI (kg/m^2^)** | 0.930 | 0.920–0.940 | <0.0001 | 0.963 | 0.952–0.974 | <0.0001 |
| **Systolic blood pressure (mmHg)** | 1.001 | 0.999–1.002 | 0.5582 |  | | |
| **Diastolic blood pressure (mmHg)** | 0.995 | 0.993–0.998 | 0.0003 | 0.995 | 0.993–0.998 | 0.0007 |
| **Hemoglobin (g/dL)** | 0.892 | 0.871–0.914 | <0.0001 | 1.015 | 0.987–1.044 | 0.2855 |
| **Fasting blood glucose (mg/dL)** | 0.999 | 0.998–1.000 | 0.0123 | 0.998 | 0.997–0.999 | <0.0001 |
| **Total cholesterol (mg/dL)** | 0.998 | 0.998–0.999 | 0.0001 | 0.999 | 0.999–1.000 | 0.198 |
| **AST (IU/L)** | 0.999 | 0.997–1.000 | 0.1360 |  | | |
| **ALT (IU/L)** | 0.991 | 0.989–0.993 | <0.0001 | 0.996 | 0.994–0.998 | 0.0002 |
| **ɤGT (IU/L)** | 0.999 | 0.999–1.000 | 0.0237 | 1.001 | 1.000–1.001 | 0.0354 |
| ALT, alanine aminotransferase; AST, aspartate aminotransferase; BMI, body mass index; CCI, Charlson’s comorbidity index; CI, confidence interval; COPD, chronic obstructive pulmonary disease; HR, hazard ratio; ɤGT, gamma-glutamyl transferase | | | | | | |

**Supplementary Table 4- Cox regression analyses for COPD development in never smokers**

| **Variable** | **Univariable analysis** | | | **Multivariable analysis** | | |
| --- | --- | --- | --- | --- | --- | --- |
|  | **HR** | **95% CI** | ***P*-value** | **HR** | **95% CI** | ***P*-value** |
| **Sex (Male vs.)** |  | | | | | |
| **Female** | 0.923 | 0.883–0.965 | 0.0004 | 0.892 | 0.840–0.946 | <0.0001 |
| **Physical activity (0/week vs.)** |  | | | | | |
| **1**–**2/week** | 0.770 | 0.728–0.814 | <0.0001 | 0.833 | 0.786–0.882 | <0.0001 |
| **3**–**4/week** | 0.675 | 0.620–0.735 | <0.0001 | 0.711 | 0.653–0.775 | <0.0001 |
| **5**–**6/week** | 0.715 | 0.616–0.830 | <0.0001 | 0.732 | 0.630–0.852 | 0.0001 |
| **Almost every day** | 1.042 | 0.960–1.130 | 0.3245 | 0.871 | 0.802–0.946 | 0.0011 |
| **Age** | 1.086 | 1.083–1.089 | <0.0001 | 1.083 | 1.079–1.086 | <0.0001 |
| **Comorbidities (CCI score)** | 1.276 | 1.253–1.300 | <0.0001 | 1.206 | 1.182–1.230 | <0.0001 |
| **BMI (kg/m^2^)** | 1.031 | 1.024–1.038 | <0.0001 | 1.016 | 1.008–1.023 | <0.0001 |
| **Systolic blood pressure (mmHg)** | 1.007 | 1.005–1.008 | <0.0001 |  | | |
| **Diastolic blood pressure (mmHg)** | 1.005 | 1.003–1.007 | <0.0001 | 0.995 | 0.994–0.997 | <0.0001 |
| **Hemoglobin (g/dL)** | 1.037 | 1.022–1.052 | <0.0001 | 1.030 | 1.011–1.051 | 0.0026 |
| **Fasting blood glucose (mg/dL)** | 1.001 | 1.000–1.001 | 0.0352 | 0.998 | 0.998–0.999 | <0.0001 |
| **Total cholesterol (mg/dL)** | 1.001 | 1.000–1.001 | 0.0069 | 0.999 | 0.998–0.999 | <0.0001 |
| **AST (IU/L)** | 1.002 | 1.002–1.003 | <0.0001 |  | | |
| **ALT (IU/L)** | 1.001 | 1.000–1.002 | 0.0048 | 1.000 | 0.998–1.001 | 0.4678 |
| **ɤGT (IU/L)** | 1.001 | 1.000–1.001 | 0.0047 | 1.000 | 1.000–1.001 | 0.5869 |
| ALT, alanine aminotransferase; AST, aspartate aminotransferase; BMI, body mass index; CCI, Charlson’s comorbidity index; CI, confidence interval; COPD, chronic obstructive pulmonary disease; HR, hazard ratio; ɤGT, gamma-glutamyl transferase | | | | | | |

**Supplementary Table 5- Baseline characteristics of male participants according to low and high ALT levels**

|  | | **Overall** | **Low ALT level group** | **High ALT level group** |  |
| --- | --- | --- | --- | --- | --- |
|  |  |  | **ALT < 40 IU/L** | **ALT ≥ 40 IU/L** | ***P*-value** |
| **Number of participants, n (%)** | | 229,095 | 185,974 (81.2) | 43,121 (18.8) |  |
| **Categorical variables, n (%)** | |  |  |  |  |
| **Smoking status** | **Never smoker** | 88,846 (38.8) | 72,989 (39.2) | 15,857 (36.8) | <0.0001 |
|  | **Ex-smoker** | 34,203 (14.9) | 27,450 (14.8) | 6,753 (15.6) |  |
|  | **Current smoker** | 95,387 (41.6) | 76,899 (41.4) | 18,488 (42.9) |  |
|  | **Unknown** | 10,659 (4.7) | 8,636 (4.6) | 2,023 (4.7) |  |
| **Physical activity** | **0/week** | 105,699 (46.1) | 85,043 (45.7) | 20,656 (47.9) | <0.0001 |
| **(number of times/week)** | **1**–**2/week** | 70,812 (30.9) | 57,058 (30.7) | 13,754 (31.9) |  |
|  | **3**–**4/week** | 25,371 (11.1) | 21,138 (11.4) | 4,233 (9.8) |  |
|  | **5**–**6/week** | 6,451 (2.8) | 5,451 (2.9) | 1,000 (2.3) |  |
|  | **Almost every day** | 13,563 (5.9) | 11,471 (6.2) | 2,092 (4.9) |  |
|  | **Unknown** | 7,199 (3.2) | 5,813 (3.1) | 1,386 (3.2) |  |
| **Continuous variables, mean ± standard deviation** | | | | | |
| **Age** | | 49.3±7.0 | 49.6±7.0 | 48.2±6.5 | <0.0001 |
| **Comorbidities** | **CCI score** | 0.3±0.7 | 0.3±0.7 | 0.3±0.8 | <0.0001 |
| **BMI (kg/m^2^)** | | 24.1±2.8 | 23.8±2.7 | 25.4±2.9 | <0.0001 |
| **Systolic blood pressure (mmHg)** | | 127.8±17.0 | 127.2±16.9 | 130.6±17.1 | <0.0001 |
| **Diastolic blood pressure (mmHg)** | | 81.3±11.4 | 80.8±11.3 | 83.2±11.5 | <0.0001 |
| **Hemoglobin (g/dL)** | | 14.9±1.1 | 14.8±1.1 | 15.1±1.1 | <0.0001 |
| **Fasting blood glucose (mg/dL)** | | 99.6±35.3 | 98.5±34.5 | 104.4±38.0 | <0.0001 |
| **Total cholesterol (mg/dL)** | | 199.6±37.7 | 197.7±36.4 | 207.7±41.6 | <0.0001 |
| **Liver function test** | **AST (IU/L)** | 28.8±19.0 | 24.9±9.1 | 45.6±34.8 | <0.0001 |
|  | **ALT (IU/L)** | 30.3±23.5 | 23.1±7.5 | 61.5±38.6 | <0.0001 |
|  | **ɤGT (IU/L)** | 50.0±61.4 | 39.7±37.1 | 94.4±108.0 | <0.0001 |
| ALT, alanine aminotransferase; AST, aspartate transaminase; BMI, body mass index; CCI, Charlson’s comorbidity index; ɤGT, gamma-glutamyl transferase | | | | | |
